# Encephalocraniocutaneous Lipomatosis: A Case Report and Literature Review

**DOI:** 10.7759/cureus.32498

**Published:** 2022-12-14

**Authors:** Deivanai Subbiah, Nur Hafidza Asiff, Norhafizah Hamzah, Amir Samsudin

**Affiliations:** 1 Ophthalmology, University of Malaya Medical Centre, Kuala Lumpur, MYS; 2 Ophthalmology, Hospital Kuala Lumpur, Kuala Lumpur, MYS; 3 Ophthalmology, Hospital Tunku Azizah, Kuala Lumpur, MYS

**Keywords:** retrobulbar mass, multiple intracranial lipomas, polymicrogyria, corneal dermoid, enchephalocraniocutaneous lipomatosis

## Abstract

Encephalocraniocutaneous lipomatosis (ECCL) or Haberland syndrome is a neurocutaneous disorder of the skin, eye, and central nervous system. A three-month-old girl was referred to our center for further management of a large left eye corneal dermoid. At birth, a small lesion was noted. Magnetic resonance imaging (MRI) around the first week of life showed an extraocular dermoid cyst measuring 1 mm x 7 mm, dysplasia of the left greater wing of sphenoid, closed-lip schizencephaly of the left parietal lobe, and polymicrogyria. During examination under anesthesia at our center, we found that the corneal dermoid had grown in size to 17 mm x 16 mm, with posterior embryotoxon, a hazy cornea, and intraocular pressure of 26 mmHg. With the anterior segment dysgenesis and secondary glaucoma, we started Gutt Timo-Comod BD. Serial MRI imaging at four months of age revealed further enlargement of the dermoid, a new left retrobulbar mass, and multiple intracranial lipomas. A diagnosis of ECCL was made at this point based on the MRI and clinical findings. A multidisciplinary meeting was held among ophthalmology, neurosurgery, radiology, and otorhinolaryngology (ORL) teams, which concluded that surgical intervention such as tumor debulking might cause more harm than benefit. Hence, she was planned to undergo close monitoring with serial MRIs and only for surgical intervention, in the presence of airway compression or any neurological deficits. The ophthalmologist should be aware of the specific radiological and clinical findings in ECCL as management of the condition would be best through a multidisciplinary approach.

## Introduction

Encephalocraniocutaneous lipomatosis, also known as Haberland syndrome, is a rare, sporadic neurocutaneous disorder with skin, ocular, and central nervous system involvement [1]. It is an ectomesodermal dysgenesis that may be caused by mosaicism for a mutation in an autosomal gene, which is responsible for encoding a factor involved in vasculogenesis and the development of mesenchymal tumors [2,3]. The most common finding in this syndrome is the presence of benign central nervous system lipomas, which are generally stable throughout a patient’s life [4].

## Case presentation

Our patient was born at 37 weeks of gestation, with spontaneous vertex delivery at a primary care center. Her mother had attended antenatal follow-ups regularly as she had gestational diabetes, placenta previa type 2, and anemia during the pregnancy. At birth, the mother noticed a small mass on the left eye. The child was seen by a pediatric ophthalmologist on day 1 of life and was noted to have a left eye corneal dermoid as well as alopecia and a subcutaneous lipoma on the frontal scalp.

She underwent an examination under anesthesia on day 5 of life, which revealed a corneal dermoid involving the entire circumference of the cornea. A contrast-enhanced computed tomography (CT) scan of the brain and orbits showed the dermoid cyst measuring 2 mm x 10 mm (anteroposterior length [AP] x width [W]), and the globe and optic nerves were intact. The radiologist proceeded with an MRI brain and orbits; this showed the dermoid cyst at the medial angle of the left eye measuring 1 mm x 7 mm (AP x W), with no extension to the posterior extraconal space. The lens and vitreous were normal, but there was also dysplasia of the left greater wing of the sphenoid, parietal closed-lip schizencephaly, and polymicrogyria. On direct examination of the oral cavity, a small fibrous alveolar soft tissue mass with a broad base about 10 mm in size was also found. The oral-maxillofacial team planned to biopsy this lesion if it increased in size.

**Figure 1:**
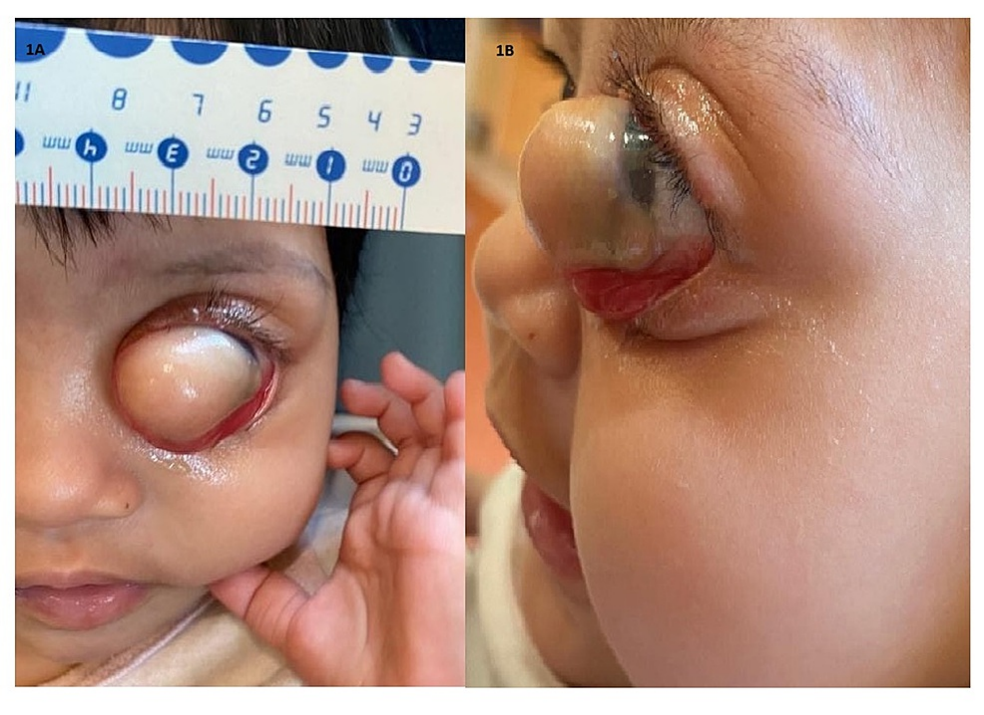
(A and B) Large corneal dermoid of the left eye covering nearly the whole cornea

At two months of age, the parents noticed that the lesion had increased in size slightly, and there was slight bleeding from the angle of the left eye due to irritation of the nasal conjunctiva. Subsequently, at three months of age, the patient was referred to our center for consideration for surgical intervention and further follow-up. Another examination under anesthesia at this point found that the corneal dermoid now measured 17 mm (horizontally) x 16 mm (vertically), covering almost the whole cornea, while another small dermoid measuring 1 mm x 1 mm was also seen temporally (Figure 1, Panels A and B). There was also posterior embryotoxon and a hazy cornea, and the intraocular pressure was 26 mmHg. The right eye findings were insignificant. With the anterior segment dysgenesis and secondary glaucoma, our patient was started on Gutt Timo-Comod BD for her left eye. We also performed left eye tarsorrhaphy as a temporary measure for lagophthalmos, which was now developing.

**Figure 2:**
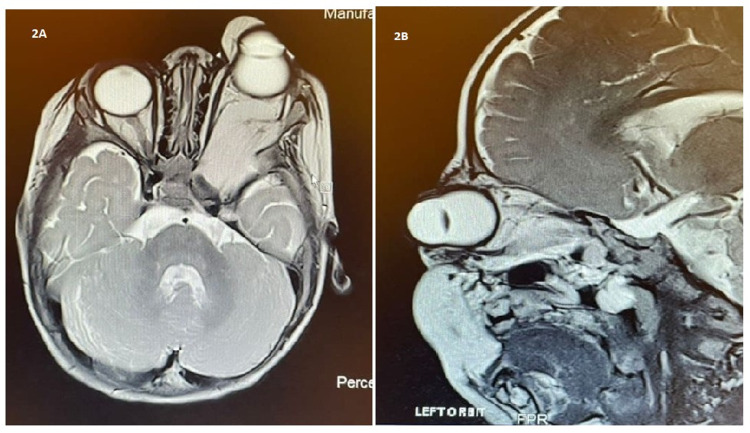
(A and B) Axial and sagittal MRI views showing left eye corneal dermoid and left-sided retrobulbar mass extending to the cavernous sinus

At four months of age, MRI revealed an enlarging limbal dermoid cyst measuring 8 mm x 17 mm (AP x W), progressively enlarging non-encapsulated fat containing a retrobulbar mass extending to the cavernous sinus and sphenopalatine area, a left frontal extra-axial intracranial lipoma with adjacent left frontal cortical polymicrogyria, a small fronto-basal extra-axial paramedian lipoma, as well as the involvement of the nasopharynx (Figure 2, Panels A and B). These lesions were supplied by branches from the anterior and middle cerebral arteries, which appeared to be dilated. The diagnosis of ECCL was made based on these MRI and clinical findings. The right eye vision was 6/24, while the left eye vision was blinking to light.

We referred our patient to the otorhinolaryngology (ORL) and neurosurgical teams. Although the airway was still patent, a flexible nasolaryngoscope examination of the left nasal cavity revealed nasopharyngeal bulging, which covered almost 90% of the posterior choana. Audiometry showed severe left-sided hearing loss. A multidisciplinary meeting was held among ophthalmology, neurosurgery, radiology, and ORL teams, which concluded that the lesion was benign but aggressive. Treatment options were discussed, including the risk of recurrence from the biopsy of the anterior part of the lesion, and it was decided that debulking surgery would be destructive and may lead to cranial nerve palsies or bleeding from carotid arteries. Hence, she was planned for close monitoring with serial MRIs. From an ophthalmology point, we decided that exenteration might be needed in the future once the growth was stable, especially for cosmetic purposes.

This patient had repeat MRI scans at six months and nine months of age. There was no significant progression at six months, while there was a minimal anteroposterior increment of the left retrobulbar encapsulated mass at nine months. Other findings were the same as preceding MRIs. Clinically, the child was active and developing well, without any episodes of seizures.

## Discussion

ECCL is a rare, sporadic, congenital neurocutaneous disorder that involves ectomesodermal tissue such as skin, eyes, and central nervous system [3]. The term was first introduced by Haberland and Perou in 1970 [1]. There is no gender or geographical preponderance, and the lesions are usually unilateral [4,5]. The pathogenesis involves a sporadic mutation to the FGFR1 gene that is involved in making a protein called fibroblast growth factor receptor that triggers signaling within cells, which in turn is important for the development of several parts of the body including the brain [6].

Although progression or late development of prosencephalic cysts, cerebrovascular aneurysms, and cystic bone lesions has been reported, the hallmark of the disease is benign central nervous system (CNS) lipomas that are generally stable throughout life [4]. In ECCL, the common location of the lipomatous mass is at the cerebellopontine angle, while it is located in the hemispheres in other diseases [2,6,7]. However, it is located in the frontal cortex in our patient, with associated polymicrogyria. Pathak et al. have previously described the presence of polymicrogyria in their report [6]. Patients can have normal development without seizures [8], as in our patient; however, there are others with normal development but with seizures, and some have mental retardation of various degrees [2]. Patients usually present with seizures in infancy or develop seizures secondary to hydrocephalus [4]. Other CNS findings include arachnoid cysts, spinal lipomas that can extend down the whole length of the spine, and tethered spinal cords due to these spinal lipomas [2,4,9].

The ocular manifestations of ECCL include dermolipomas, corneoscleral anomalies, anterior chamber abnormalities, ocular or palpebral colobomas, aniridia, microphthalmia, and calcification of the globe [2]. Vascular abnormalities such as coarctation of the aorta are rare but highly specific for ECCL [2]. Nevus psiloliparus, a well-demarcated, alopecic fatty tissue nevus on the scalp, is the most characteristic skin anomaly seen in ECCL, although our patient did not have it [2]. Granular mucosa visible on the surface of tongues is another finding that has also been described [8].

Complications also include those related to CNS malformations. There is a risk of neoplasms such as juvenile extra-nasopharyngeal angiofibromas of gingiva, jaw tumors, papillary glioneuronal tumors, and low-grade gliomas/astrocytomas [2,3]. The jaw tumors might be progressive, whereas the osteomas, lipomas, choristomas, and adenomas are present at birth and usually do not progress [2]. Our patient has loss of hearing on the left side due to the extension of the tumor to the sphenopalatine area. She also has an alveolar soft tissue mass, which needs maxillofacial follow-up to observe for progression.

Our patient fits in with the diagnosis of ECCL as shown in Tables 1, 2. Although she does not have nevus psiloliparus, she has major criteria in three systems that include the corneal dermoid of the eye (choristoma), intracranial lipoma of the CNS and alveolar fibrous soft tissue mass, and also two minor skin criteria (patchy non-scarring alopecia and subcutaneous lipoma in frontotemporal region).

**Table 1:** Revised diagnostic criteria for encephalocraniocutaneous lipomatosis (ECCL) by Moog (2009) Source: Ref. [2].

System	Major Criteria	Minor Criteria
Eye	1. Choristoma, with or without associated anomalies	1. Corneal and other anterior chamber anomalies
		2. Ocular or eyelid coloboma
		3. Calcification of globe
Skin	1. Proven nevus psiloliparus (NP)	1. Possible NP
	2. Possible NP and ≥1 of minor criteria 2–5	2. Patchy or streaky non-scarring alopecia (without fatty naevus)
	3. ≥2 of minor criteria 2–5	3. Subcutaneous lipoma(s) in frontotemporal region
		4. Focal skin aplasia/hypoplasia on the scalp
		5. Small nodular skin tags on eyelids or between the outer canthus and tragus
CNS	1. Intracranial lipoma	1. Abnormal intracranial vessels, e.g., angioma and excessive vessels
	2. Intraspinal lipoma	2. Arachnoid cyst or other abnormality of meninges
	3. ≥2 of minor criteria	3. Complete or partial atrophy of a hemisphere
		4. Porencephalic cyst(s)
		5. Asymmetrically dilated ventricles or hydrocephalus
		6. Calcification (not basal ganglia)
Others	1. Jaw tumor (osteoma, odontoma, or ossifying fibroma)	
	2. Multiple bone cysts	
	3. Aortic coarctation	

**Table 2:** Application of criteria to the diagnosis of ECCL ECCL: Encephalocraniocutaneous lipomatosis; NP: Nevus psiloliparus.

Definite Case
1. 3 systems involved, major criteria in ≥2, or
2. 3 systems involved, proven NP or possible NP + ≥1 of minor skin criteria 2–5
3. 2 systems involved with major criteria, one of which proven NP or possible NP + ≥1 of minor skin criteria 2–5
Probable Case
1. 2 systems involved, major criteria in both
2. 2 systems involved, proven or possible NP

The differential diagnosis for her condition includes proteus syndrome, oculoectodermal syndrome, oculocerebrocutaneous syndrome, Goltz syndrome, and Goldenhar syndrome [2,5]. Proteus syndrome has a progressive course and includes cerebriform connective tissue naevus, while ECCL is not progressive [2,7]. Hyperostosis of the skull, dysregulation of adipose tissue, and vascular malformations are part of both syndromes. Oculoectodermal syndrome might be a mild variant of ECCL with a lack of intracranial lipomas [2]. Oculocerebrocutaneous syndrome is similar to ECCL but does not present with nevus psiloliparus; instead, these patients present with a post-auricular almond-shaped hypoplastic skin defect [2]. The Goltz syndrome is rare and consists of a triad of various linear nevi, epilepsy, and mental retardation [5]. Meanwhile, Goldenhar syndrome consists of epibulbar choristomas and systemic abnormalities affecting the cardiac, genito-urinary, and pulmonary systems [5].

So far, there is no effective treatment for ECCL. Ophthalmic management may include excision of the choristoma, lamellar keratoplasty, and visual rehabilitation [3]. There have been patients who underwent ventriculoperitoneal shunts for hydrocephalus [4]. Follow-up is important to look out for progression, conversion to neoplasia, or other complications. If there is airway compression, the lipoma threatens vital structures such as the internal carotid artery or the brain, or there are any neurological deficits, it is an indication for tumor debulking surgery.

As the child grows, she will need psychosocial support as the tumor can be disfiguring. Not only are they able to provide psychosocial support, but they also try to improve the quality of life for patients. Finally, this child might also need to attend a special needs school as she has both visual and hearing impairment on her left side.

## Conclusions

Encephalocraniocutaneous lipomatosis is a neurocutaneous syndrome with a specific set of radiological and clinical findings. The ophthalmologist has to be vigilant as the eye findings might precede other clinical findings. Early recognition, early imaging, and a multidisciplinary approach to treatment may not only ensure a better quality of life for patients but even increase the life expectancy of patients. More case reports and case series are necessary to understand the nature of this disease and to explore the treatment options that are available for these patients.
